# Cortical thickness lateralization and its relation to language abilities in children

**DOI:** 10.1016/j.dcn.2019.100704

**Published:** 2019-08-22

**Authors:** Ting Qi, Gesa Schaadt, Angela D. Friederici

**Affiliations:** a Department of Neuropsychology, Max Planck Institute for Human Cognitive and Brain Sciences, Leipzig, Germany; b Clinic of Cognitive Neurology, Medical Faculty, University Leipzig, Germany; c Department of Neurology, Max Planck Institute for Human Cognitive and Brain Sciences, Leipzig, Germany

**Keywords:** Language development, Sentence comprehension, Structural asymmetry, Cortical thickness, Longitudinal study

## Abstract

The humans’ brain asymmetry is observed in the early stages of life and known to change further with age. The developmental trajectory of such an asymmetry has been observed for language, as one of the most lateralized cognitive functions. However, it remains unclear how these age-related changes in structural asymmetry are related to changes in language performance. We collected longitudinal structural magnetic resonance imaging data of children from 5 to 6 years to investigate structural asymmetry development and its linkage to the improvement of language comprehension abilities. Our results showed substantial changes of language performance across time, which were associated with changes of cortical thickness asymmetry in the triangular part of the inferior frontal gyrus (IFG), constituting a portion of Broca’s area. This suggests that language improvement is influenced by larger cortical thinning in the left triangular IFG compared to the right. This asymmetry in children’s brain at age 5 and 6 years was further associated with the language performance at 7 years. To our knowledge, this is the first longitudinal study to demonstrate that children’s improvement in sentence comprehension seems to depend on structural asymmetry changes in the IFG, further highlighting its crucial role in language acquisition.

## Highlights

• Language development and the brain’s asymmetry investigated between 5 and 6 years.
• Substantial language improvements in children across development.
• Higher language improvement associated with stronger IFG asymmetry changes.
• The IFG’s asymmetry is further predictive of language abilities at age 7 years.
• Results highlight the crucial role of the IFG in language acquisition.

## 1 Introduction

The human brain is anatomically and functionally asymmetric. This so-called brain lateralization has been observed for various cognitive functions (Toga and Thompson, 2003), such as language, favoring the left hemisphere in the healthy adult brain. The lateralization of language-related regions has been revealed in the brain’s cortical thickness, supposed to be derived from different thinning rates across hemispheres during development (Shaw et al., 2009; Zhou et al., 2013). Specifically, a thinner cortex has frequently been observed in left-hemispheric language-related regions compared to their right counterparts [for example the IFG (Luders et al., 2006; Zhou et al., 2013)], although an equivocal picture remains concerning the direction of the observed asymmetry (Kong et al., 2018; Plessen et al., 2014; Zhou et al., 2013). That language abilities are related to the brain’s asymmetry becomes especially apparent when looking at brain asymmetry of individuals with language difficulties, such as developmental dyslexia (Altarelli et al., 2014; Bishop, 2013; Qi et al., 2016), assumed to rely on a phonological processing deficit. In addition, because atypical brain asymmetry is already present in individuals with a familial risk of dyslexia during their early childhood (Vanderauwera et al., 2018), it could be suggested that atypical brain asymmetry is one of the causes of this developmental disorder.

Concerning the developmental pattern of the brain’s asymmetry, structural asymmetry and functional lateralization seem to be present already in the fetus (Dehaene-Lambertz et al., 2006; Dubois et al., 2010; Habas et al., 2012; Kasprian et al., 2011), but both structural and functional asymmetries were shown to further evolve, particularly during childhood and adolescence (Minagawa-Kawai et al., 2011; Shaw et al., 2009; Zhou et al., 2013). The asymmetry of cortical thickness has been reported to change with age from childhood to adulthood (Kong et al., 2018; Plessen et al., 2014; Zhou et al., 2013). Specifically, focusing on language-related regions, the IFG was shown to become more right-lateralized (i.e., greater thinning in the left compared to the right hemisphere) with increasing age (Shaw et al., 2009; Zhou et al., 2013), while the superior temporal gyrus (STG) and the temporo-parietal junction were shown to become more left-lateralized (i.e., less thinning in the left compared to the right hemisphere) with age (Kong et al., 2018; Zhou et al., 2013). Next to structural changes, the brain’s functional asymmetry, possibly influenced by the brain’s structural asymmetry, was also shown to substantially change throughout development. A functional leftward asymmetry in language-related regions (e.g., Broca’s area in the IFG) was shown to become stronger from infancy to toddlerhood (Minagawa-Kawai et al., 2011), with a further increase up to the age of 20 years (Szaflarski et al., 2006). Moreover and importantly, the brain’s structural asymmetry and functional asymmetry have been suggested to be related to language abilities. Cross-sectional studies have shown that children with larger vocabulary demonstrate a stronger leftward white matter asymmetry in the arcuate fasciculus [AF, connecting Broca’s area in the inferior frontal cortex with Wernicke’s area in the temporal cortex (Lebel and Beaulieu, 2009)], and show a stronger leftward functional asymmetry for a language production task (Groen et al., 2012). Thus, the anatomical and functional layout of the language-related brain regions are asymmetric and, more crucially, change with increasing language abilities.

Despite the broad interest in the development of the brain’s asymmetry and its association with language abilities, there are only a few longitudinal studies that examined the developmental trajectory of the brain’s asymmetry. Following children from birth to 2 years of age, Li et al. (2015) found cortical thickness asymmetries to change gradually during the first 2 years of life. Moreover, a group of children followed from childhood to early adulthood showed cortical thickness increase in the left compared to the right hemisphere in temporo-occipital regions and cortical thickness reduction in the left compared to the right hemisphere in anterior regions, including orbitofrontal and inferior frontal regions (Shaw et al., 2009). However, the linkage between longitudinal changes of the brain’s cortical thickness asymmetry and cognitive abilities, in particular language abilities, still remains largely elusive. Specifically, sentence comprehension - a more advanced language ability requiring syntactic knowledge which emerges later in development compared to other language domains (e.g., phonology, vocabulary) - has hardly been investigated in the context of the brain’s cortical thickness asymmetry. It has been suggested that the relatively slow acquisition of sentence comprehension goes along with the slow functional development of the IFG and its white matter connectivity to the posterior temporal cortex (Skeide and Friederici, 2016). Indeed, it has been shown that sentence comprehension abilities were correlated with the brain’s functional maturation in the language-related regions and with the brain’s structural maturation of language-related connections in children between the ages of 3 and 10 years (Schipke et al., 2012; Skeide et al., 2016). However, sentence comprehension, which is highly relevant for a child’s successful language acquisition and essential for social communication (Kuhl et al., 2005), has rarely been investigated from the perspective of cortical thickness and its asymmetry.

Thus, in the present study we aimed to investigate the development of the brain’s structural asymmetry in young children and its linkage to changes of language abilities. Specifically, sentence comprehension was examined in relation to the brain’s structural asymmetry. To do so, we collected structural magnetic resonance imaging (sMRI) data and tested sentence comprehension abilities of 5-year-old children. These children were then re-invited 1) a year later (i.e., at age 6), where we again collected their sMRI data and sentence comprehension abilities, and 2) two years later, where we again tested their sentence comprehension abilities (i.e., at age 7). We specifically decided to test the children at this age range, because this developmental window is typically characterized by advancing development in brain function and structure (Gogtay et al., 2004; Knoll et al., 2012; Skeide et al., 2014), as well as by steady changes in language abilities (Sakai, 2005), in particular, sentence comprehension (Lindner, 2003). We first delineated brain asymmetry patterns by calculating the cortical thickness asymmetry of the language-related regions for each sMRI testing time point (i.e., at age 5 and at age 6) to then analyze the developmental trajectory of cortical thickness asymmetry and sentence comprehension abilities across time. In a next step, we investigated how the individual changes in structural asymmetry and the individual changes in language abilities covary. Finally, we correlated the brain’s asymmetry at 5 and 6 years with these children’s later language abilities, tested when they were 7 years. Given previous data on longitudinal changes in cortical thickness asymmetry evidenced as asymmetry changes in the anterior and temporal brain regions (Li et al., 2015; Shaw et al., 2009) and the independently reported increase in language abilities across development (Sakai, 2005; Schipke et al., 2012), we expected longitudinal changes in structural asymmetry primarily in the frontal and temporal language-related brain region. Moreover, we hypothesized that the individual development of structural asymmetry in anterior and temporal language-related brain regions (Li et al., 2015; Shaw et al., 2009; Zhou et al., 2013) is associated with language abilities, and, crucially, that individual changes in structural asymmetry are coupled with changes in language abilities across development. We further expected that the structural asymmetry of the young brain (i.e., at age 5 and 6) is predictive for later language performance (i.e., at age 7).

## 2 Methods

### 2.1 Participants

In total, 76 children (38 girls) were initially invited to participate in the present study. Twenty out of these 76 children were excluded due to the following reasons: 1) Did not pass the data quality control as described below (N = 11) and 2) Did not fulfill criteria for being right-handers (N = 9). The reason for only including right-handers was because of the high possibility (i.e., ˜ 30%) of atypical functional lateralization in left-handers (Knecht, 2000; Knecht et al., 2000). Handedness was determined by the modified version of the Edinburgh Handedness Inventory (Oldfield, 1971). An adjustment was justified due to the fact that some of the items were not suitable for children (e.g., using a broom, writing a letter), which were excluded from the Inventory in the present study. This modified version has been frequently and successfully used in previous studies (Skeide et al., 2016; Wu et al., 2016), resulting in a continuous score as an indicator of the extent of handedness. After exclusion, a total of 56 children (26 girls) were included in the present study. Detailed information regarding the sample inclusion can be found in Figure S1 in the supplements. At the first testing, their *mean* age was 5.47 years [standard deviation (*SD*) = 0.28] and at the second testing, their *mean* age was 6.47 (*SD* = 0.27), with a *mean* interval of 11.97 months (*SD* = 1.22) between the two testing time points. The third testing time point, where we only assessed the children’s sentence comprehension abilities, took place when children were 7.62 years old (*SD* = 0.33). The *mean* interval between the second and third testing time point was 13.77 months (*SD* = 3.23) (see Table 1). Non-verbal IQ was assessed when children were 5 years of age using the language-independent scale of the German version of the Kaufman Assessment Battery for children (K-ABC, Kaufman and Kaufman, 1983). Participants showed a *mean* non-verbal IQ of 107.11 (*SD* = 9.45) ranging from 88 to 126 (i.e., within or above the normal range from 85 to 115). All participants were native German speakers, with no history of medical, psychiatric or neurological disorders. Written informed consent was obtained from the legal guardian or parents of the children, and children gave verbal assent for attendance before the experiments. The ethical review board of the University of Leipzig approved the study.

**Table 1:** Descriptive statistics of the children for all three testing time points separately.

Time points	Brain assessment		Behavior assessment: TSVK scores		
	N (M/F)	Age	N (M/F)	Age	Raw scores
Time point 1	56 (30/26)	5.47 (0.28)	55 (29/26)	5.48 (0.28)	24.95 (2.99)
Time point 2	56 (30/26)	6.47 (0.27)	52 (29/24)	6.46 (0.27)	28.71 (2.77)
Time point 3	–	–	51 (28/23)	7.62 (0.33)	30.47 (2.61)

*Note*: Children without behavioral data (time point 1: N = 0, time point 2: N = 3, time point 3: N = 5) and with behavioral scores but detected as outliers (time point 1: N = 1, time point 2: N = 1, time point 3: N = 0) were excluded when reporting the mean scores. Numbers in brackets indicate standard deviations.

### 2.2 Behavioral language test

A standardized language test [Test zum Satzverstehen von Kindern, TSVK, (English: sentence comprehension test for children), Siegmüller et al., 2011] was administered, assessing the general sentence comprehension abilities of children. The TSVK employs a picture matching task, in which the child is auditorily presented with a sentence as well as with three pictures. The child’s task is to choose the correct picture matching the presented sentence. In total, the child is presented with 36 items that vary in sentence complexity, which was manipulated by word order, tense, mode, clause number, pronoun type, and verb type. The number of correct responses gets summed (raw scores) and converted to standard *T* scores. Of note and important for the present study, the age-standardized *T* scores would remove the time trend (i.e., developmental information) from longitudinal data (Bollen and Curran, 2006). Therefore, we only present findings on the raw score of the TSVK in the following.

### 2.3 Data acquisition

T1-weighted MRI data were collected on a Siemens 3 T MRI scanner with a 12 channel array head coil. The T1-weighted magnetization prepared gradient-echo (MP-RAGE) image was acquired with the following parameters: TI = 740 ms; TR =1480 ms; TE =3.46 ms; alpha = 10°; image matrix = 256 × 240; FOV = 256 × 240 mm^2^; 128 sagittal slices; spatial resolution = 1 × 1 × 1.5 mm^3^. Children were asked to participate in a mock scan before the formal MRI scanning to familiarize with the environment and the experimental procedure.

### 2.4 Data processing

Before processing, all T1-weighted images were visually inspected for potential blurring, ringing, striping, ghosting, etc. caused by head motion during scanning, to ensure that brain tissues can be well-differentiated. Those with severe problems on the raw images were considered with caution or excluded. Further, the image quality was checked using the Computational Anatomy Toolbox (CAT 12, for more details, see http://dbm.neuro.uni-jena.de/vbm/check-sample-homogeneity) and only those within the recommended criteria entered the following preprocessing (i.e., one child was excluded due to bad raw image quality at the second testing time point). Cortical reconstruction and volumetric segmentation were performed using Freesurfer (v 6.0.0), (http://surfer.nmr.mgh.harvard.edu/). To obtain reliable volume and cortical thickness estimates, images were automatically processed with the longitudinal stream (Reuter et al., 2012). Specifically, the unbiased within-subject template space and image (Reuter and Fischl, 2011) was created using the robust, inverse consistent registration (Reuter et al., 2010). Several procedures, such as skull stripping, Talairach transforms, atlas registration, as well as spherical surface maps and parcellations were then initialized with common information from the within-subject template, to increase reliability and statistical power (Reuter et al., 2012). The reconstructed surfaces were visually inspected and manually edited for inaccuracies (e.g., pial surface and white matter segmentation errors, intensity normalization errors) for all intermediate (i.e., process individual data at all time points cross-sectionally and create a subject-specific template from each time point) and final runs (i.e., process all time points longitudinally). The most commonly observed inaccuracy in our sample was misclassification of pial surface in a few vertices around membranes and vessels adjacency and was fixed, for example, by editing a few vertices on the *brainmask*. For a similar procedure, see Cafiero et al. (2018) and for detailed information of guidelines for manual editing, see Freesurfer troubleshooting tutorials (https://surfer.nmr.mgh.harvard.edu/fswiki/FsTutorial/LongitudinalTutorial). Surfaces that had been manually edited were regenerated by rerunning the automated Freesurfer pipeline and re-inspected (i.e., 10 children were excluded from the longitudinal analysis due to the failure of surface reconstruction or parcellation: 10 failed at the first testing point and 8 out of 10 also failed at the second time point, see Fig. S1 in the supplements). The cortical thickness was calculated as the closest distance from the pial surface to the white matter surface at each vertex (Fischl and Dale, 2000).

### 2.5 Laterality index calculation

The laterality index, quantifying the cortical thickness difference between left and right hemisphere, was calculated as:

Laterality index = (*L - R*)/(*L + R*)

where *L* refers to the cortical thickness of the left hemisphere, and *R* refers to the cortical thickness of the right hemisphere. The laterality index ranges from -1 to 1, where -1 indicates a complete right-lateralized brain region and +1 indicates a complete left-lateralized brain region. We computed both regional- and vertex-based laterality indices. At regional-level, we selected several language-related regions as regions of interest (ROIs) based on the *Desikan-Killiany* parcellation (Desikan et al., 2006) implemented in Freesurfer. These language-related ROIs included temporal and parietal regions these are the bankssts [i.e., banks of the superior temporal sulcus (STS)], superior temporal gyrus (STG), middle temporal gyrus (MTG), supramarginal gyrus (SMG), transverse temporal [i.e., Heschl’s gyrus, (HG)], temporal pole (TP), inferior parietal cortex (IPC), and frontal regions namely the caudal middle frontal gyrus (i.e., caudal part of MFG), pars opercularis (i.e., opercular part of the IFG), pars triangularis (i.e., triangular part of the IFG), pars orbitalis (i.e., orbital part of the IFG). Note that these three latter parts of the IFG are also known in the Brodmann (BA)-based terminology as BA 44, BA 45 and BA 47, with BA 44 and BA 45 making up classical Broca’s area. This automatic parcellation, based on the hemispheric-specific anatomical features, has been discussed to show high accuracy compared to manual labeling (Desikan et al., 2006). We additionally inspected the parcellation for each subject visually to ensure its accuracy. The ROIs covered most of the frontal and temporal regions, including inferior, middle frontal, superior temporal, middle temporal cortices, inferior parietal lobule, and supramarginal gyrus, which have been reported as core language regions in the previous literature (Fedorenko and Thompson-Schill, 2014; Friederici, 2012; Friederici and Gierhan, 2013; Vigneau et al., 2006). Furthermore, we performed a term-based meta-analysis in NeuroSynth (Yarkoni et al., 2011, http://neurosynth.org/) using terms such as language comprehension, sentence comprehension, and language comprehension network to confirm the selection of ROIs (see supplements and Fig. S2). In addition, to obtain a complete overview with respect to the cortical thickness asymmetry of the whole brain, we examined the asymmetry pattern at the whole brain level for all the parcellations from the *Desikan-Killiany* atlas (see supplements). To cancel out any influence from ROI selection using a specific atlas (e.g., number of regions defined in the atlas), we performed an additional vertex-based analysis, considering both hemisphere equally for each vertex (Fonov et al., 2011; Greve et al., 2013), for validation purposes (see supplements).

### 2.6 Statistical analyses

Before the main analysis, we checked for the outliers concerning the laterality index and the raw scores of TSVK using the criterion of 3 median absolute deviations from the median (Leys et al., 2013) for each age group (see Fig. S1). No outliers were identified for the laterality index neither at the first nor at the second testing time point. Further, no outlier was identified when analyzing the change of laterality across the two testing time point (i.e., change of laterality between the age of 5 and 6 years). However, when examining the raw scores of the TSVK, one subject was identified as an outlier at time point one (i.e., raw score = 14) and one subject was identified as an outlier at time point two (i.e., raw score = 20). Please note that these two children were also identified as outliers when analyzing the change of TSVK scores across the two time points. For the third testing time point, at the age of 7.62, we did not identify any outliers when analyzing the TSVK scores. First, we examined the direction of asymmetry for each brain region by conducting *one sample t-tests* on the laterality index to examine whether the laterality index differed significantly from zero when children were 5 years, as well as when they were 6 years old. Then, the *nlme* package (Pinheiro et al., 2007) for linear mixed-effect models implemented in R was applied to analyze our longitudinal data. For all models, a fixed effect of time and a random effect of subject were examined, and baseline age (i.e., age at first testing time point), and gender were controlled for by including them as covariates of no interest. Of note, even though we only included right-handers, the continuous variable handedness was still considered as a covariate of no interest to control for potential influences. When analyzing the development of language abilities over time, the autoregressive covariance structure, which is commonly used in longitudinal studies with equally spaced time intervals for each measurement, was assumed for models with multiple repeated measures (Arnau et al., 2010). Meanwhile, non-verbal IQ, together with the covariates mentioned above, was included as a covariate of no interest when performing analyses concerning language abilities. To assess the relationship between brain asymmetry and language performance, we correlated brain asymmetry with language performance. Specifically, to examine how changes in brain asymmetry covary with changes in sentence comprehension abilities, the difference scores (i.e., brain asymmetry and sentence comprehension abilities changes) were calculated by subtracting the measures at the first time point from the measures at the second time point. Similarly, the difference scores for sentence comprehension abilities changes between the second time point and the third time point were obtained. Larger values indicate greater language improvements or greater increase towards stronger leftward cortical thickness asymmetry, whereas smaller values would indicate relatively smaller language changes or greater rightward asymmetry. Bonferroni correction was used for multiple comparisons with a significance level of *p* <  0.005 (i.e., 0.05/11 ROIs).

## 3 Results

### 3.1 Longitudinal language performance changes

We found a significant fixed effect of time (estimate = 2.80, *t* (101) = 3.66, confidence interval (*CI*): 2.40–3.20, *p* <  0.001), indicating that language abilities changed over time. Meanwhile, the *SD* of the intercept was 1.68 with a *CI* between 1.20 and 2.36, suggesting that the intercept of the random effect was significant, meaning that there were large individual differences in language performance. We also introduced the random slope to the model, however it did not improve the model significantly (see supplements Table S1), suggesting that there were similar growth patterns across different subjects. The detailed descriptive statistics on sentence comprehension abilities (i.e., TSVK) are shown in Table 1.

### 3.2 Longitudinal cortical thickness asymmetry changes

**Fig. 1:**
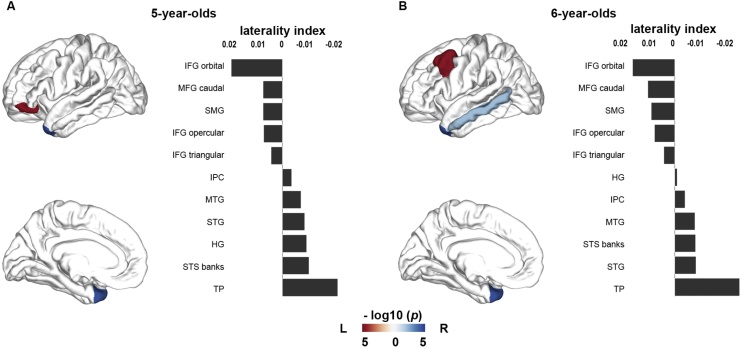
Language-related cortical thickness asymmetry patterns in children aged 5 years (A) and 6 years (B). A positive laterality index indicates leftward asymmetry, while a negative laterality index indicates rightward asymmetry. The left panel of each figure shows brain regions with significant asymmetries (*one-sample t-test*; *p* < 0.005, Bonferroni-corrected). Red colored regions of interest (ROIs) denote significant left-lateralized regions, and blue colored ROIs denote significant right-lateralized regions. The right panel of each figure illustrates laterality indices of all ROIs ordered by the strength of the mean laterality indices. Note at 6 years (B), due to a large standard deviation, IFG orbital with the largest mean laterality index (in the right panel) did not show significant asymmetry as illustrated in the left panel. For visualization purposes, *p*-values were log-transformed and a darker color denotes more significant *p*-values. L = left; R = right (For interpretation of the references to colour in this figure legend, the reader is referred to the web version of this article).

We next examined the cortical thickness asymmetry pattern of the children when they were 5 years and when they were 6 years old, separately. At age 5, the orbital part of the IFG showed significant leftward asymmetry (i.e., thinner in the right hemisphere compared to left; *p* <  0.005, Bonferroni corrected), and the temporal pole showed significant rightward asymmetry (i.e., thinner in the left hemisphere compared to right; *p* <  0.005, Bonferroni corrected, see Fig. 1A). Several regions showed a trend for significance (*p* <  0.05, uncorrected, see Table S2 in the supplements), including a leftward asymmetry of the opercular part of the IFG, of the SMG and of the caudal part of the MFG, and a rightward asymmetry of the MTG, of the STG and of the banks STS. To obtain a complete overview of the asymmetry, the asymmetry patterns of the entire brain at both, regional- and vertex-level can be found in the supplements (see supplements, Fig. S3 and Table S2). A similar asymmetry map was observed in children at age 6 (see Fig. 1B and Table S2), except that the MFG now showed significant leftward asymmetry, the MTG now showed significant rightward asymmetry (*p* <  0.005, all Bonferroni corrected) and that the orbital part of the IFG was no longer significant leftward asymmetry.

Next, we examined the longitudinal structural asymmetry changes across development. We found a fixed time effect derived from the mean asymmetry change at group-level across time in the HG, suggesting a significant asymmetry change from age 5 to 6 years in the HG. Specifically, the HG became less right-lateralized (*mean* at age 5 = −0.01, *SD* = 0.05; *mean* at age 6 = 0.00, *SD* = 0.04; *t* (55) = 3.15, *p* = 0.003, *CI*: 0.003–0.013). Further, we verified that this reduction in HG’s rightward asymmetry was driven by a more rapid thinning in the right HG compared to the left HG by post-hoc performing *paired-sample t-tests* to compare the cortical thickness between the two assessments separately for the right and left HG (see Table S6 in the supplements). In addition, the intercept for the random effect was significant for all ROIs (see supplements Table S3), implying that the brain’s asymmetry differs significantly at the individual level.

### 3.3 Associations between changes in language performance and brain asymmetry

**Fig. 2:**
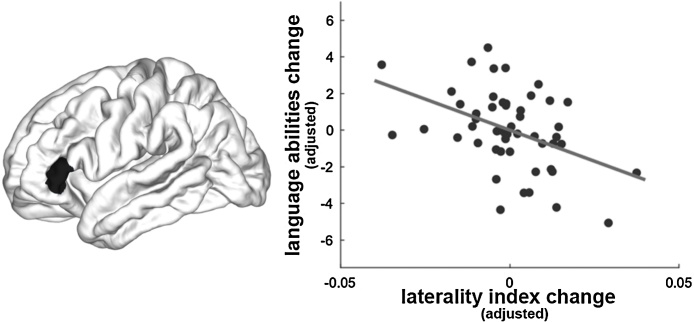
Illustrated is the negative correlation between cortical thickness asymmetry changes in the triangular part of the IFG (colored in black) and changes of language performance in children between the ages of 5 and 6 years. Of note, the x-axis indicates the changes of the laterality index across time adjusted for covariates of no interest, namely baseline age, gender, non-verbal IQ, and handedness; the y-axis indicates the changes in language abilities (i.e., TSVK raw scores) adjusted for covariates of no interest.

We examined the association between language performance and cortical thickness asymmetry separately for each time point, i.e., when they were 5 and 6 years old, however, we did not find any significant correlation for neither time point. Further, despite the minimal group-level asymmetry changes across time, asymmetry changes may emerge at the individual level. Thus, we concentrated on individual differences by correlating individual changes in brain asymmetry across time for all language-related ROIs with the individual changes in language performance. A significant negative correlation was revealed between the change in language performance and the cortical thickness asymmetry change in the triangular part of the IFG (i.e., *r* = −0.45, *p* = 0.002, Bonferroni corrected, see Fig. 2 and Table S4 in the supplements), while controlling for covariates of no interest (i.e., baseline age, gender, handedness, and non-verbal IQ). The effect remained significant when we additionally included initial language performance and initial mean cortical thickness asymmetry of the whole brain (*r* = −0.43, *p* = 0.003, Bonferroni corrected) as covariates. It suggests that greater rightward asymmetry in the triangular IFG from 5 to 6 years was associated with larger improvement in the children’s sentence comprehension abilities regardless of their initial behavioral and brain status. Further, we verified that this rightward asymmetry was driven by a more rapid thinning in the left triangular IFG compared to the right triangular IFG by post-hoc performing *paired-sample t-tests* to compare the cortical thickness between the two assessments separately for the left and right triangular part of the IFG (see Table S6 in the supplements). Performing vertex-wise analysis revealed similar results (see supplements and Fig. S4). In addition, we correlated the changes in asymmetry from age 5 to 6 years with the change in language performance from age 6 to 7 years, but we only found a trend-level negative correlation (not surviving multiple comparison correction) for the STS (*r* = −0.32, *p* = 0.036, uncorrected) and the TP (*r* = −0.35, *p* = 0.020, uncorrected), while controlling for baseline age, gender, non-verbal IQ, handedness, initial (at age 5) language performance, and initial mean cortical thickness asymmetry of the whole brain. Even though these correlations do not become significant, findings might suggest that children with greater asymmetry change to a rightward asymmetry in the STS and TP from 5 to 6 years tend to show a larger improvement in sentence comprehension from 6 to 7 years.

### 3.4 Associations between cortical thickness asymmetry and later language performance

**Fig. 3:**
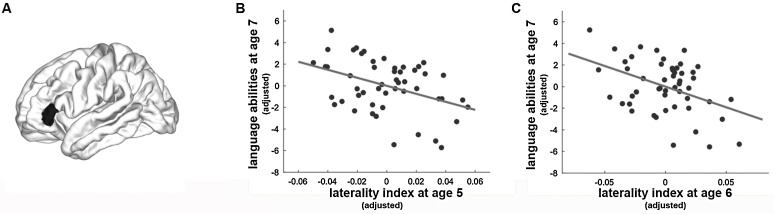
Illustrated are the associations between early brain asymmetry and later sentence comprehension abilities. Cortical thickness asymmetry of the triangular part of the IFG (A, colored in black) in children at age 5 years (B) and age 6 years (C) was negatively correlated with language abilities at age 7 years. Of note, the x-axes in (B) and (C) indicate the laterality index of children aged 5 and aged 6 years, respectively, after adjusting for covariates of no interest (i.e., baseline age, gender, non-verbal IQ, handedness). The y-axes indicate language abilities (i.e., raw scores of the TSVK) of children when they were 7 years old, after adjusting for the covariates of no interest.

Given that we additionally collected language scores of the children when they were 7 years old, we further examined associations between early cortical thickness asymmetry (at age 5 and at age 6 years) and later language performance (at age 7 years). We found that the language performance in 7-year-old children was negatively correlated with the cortical thickness asymmetry in the triangular part of IFG at age 5 years (*r* = -0.40, *p* =  0.005, Bonferroni corrected, see Fig. 3B and Table S5 in the supplements) and at age 6 years (*r* = -0.42, *p* =  0.003, Bonferroni corrected, see Fig. 3C and Table S5 in the supplements), while controlling for covariates of no interest (i.e., baseline age, gender, handedness, and non-verbal IQ), suggesting that children with superior language performance at age 7 showed lower cortical thickness in the left compared to the right triangular part of the IFG already at ages 5 and 6.

## 4 Discussion

In order to gain a better understanding of the role the brain’s structural asymmetry plays in human language development, the present study examined to what extent changes in cortical thickness asymmetry of language-related brain regions is related to language abilities across development. We found significant leftward asymmetry in the orbital part of the IFG and rightward asymmetry in the temporal pole at age 5 years, along with a similar asymmetry pattern in 6-year-old children (i.e., one year later). As expected, we also found a significant asymmetry change between the ages of 5 and 6 in the HG, but not in the frontal regions. However, individual changes in language abilities were associated with structural asymmetry changes in the triangular part of the IFG, indicating a stronger reduction in cortical thickness in the left compared to the right IFG between the ages of 5 and 6 to be associated with greater language improvement. Furthermore, greater cortical thinning in the left compared to the right triangular part of the IFG in children both, at the age of 5 and 6 years were associated with superior language performance in the very same children at the age of 7. Thus, we showed that the amount of changes in the IFG’s cortical thickness asymmetry impacts the strength of language comprehension improvement, as children with a stronger IFG asymmetry (triangular part), as well as with stronger change in the IFG’s asymmetry (triangular part) between the age of 5 and 6 years developed superiorly in their language abilities, compared to children with less asymmetry and less asymmetry change.

### 4.1 Children’s changes in language abilities across development

In line with previous research suggesting language functions to steadily increase (Dittmar et al., 2008; Sakai, 2005; Schipke et al., 2012; Skeide et al., 2016; Skeide and Friederici, 2016), we found an improvement in sentence comprehension abilities in children from 5 to 7 years. A recent model suggests that advances in language performance up to the age of 3 years are based on bottom-up processes supported mainly by the temporal cortex whereas language performance after the age of 3 years additionally involved top-down processes supported by the inferior frontal cortex (Skeide and Friederici, 2016). Here, we extended this view by showing that the improvement of language abilities between the ages of 5 and 6 years depends not only on structural changes in the IFG, but moreover on the brain’s structure asymmetry and its change across development. Before discussing our findings on the found brain-behavior association, we will first discuss our cortical thickness asymmetry findings and its change across development.

### 4.2 Children’s changes in cortical thickness asymmetry across development

Concerning cortical thickness asymmetry in children, we demonstrated a trend towards leftward asymmetry in the frontal and inferior parietal language-related regions and a trend towards rightward asymmetry in most of the temporal ROIs at both time points (i.e., at age 5 and 6). Our findings (i.e., at both regional- and vertex-level, see supplements) are in agreement with previous cortical thickness asymmetry studies, demonstrating the anterior cortex (lateral, medial, dorsal frontal regions) to be significantly left-lateralized and the posterior cortex (lateral, medial parts of posterior temporal, parieto-occipital regions) to be prominently right-lateralized (Kong et al., 2018; Plessen et al., 2014). However, the inferior frontal cortex, which was significantly left-lateralized in our children at the age of 5, but not at the age of 6 years, showed an equivocal picture across the literature (Koelkebeck et al., 2014; Kong et al., 2018; Luders et al., 2006; Plessen et al., 2014; Shaw et al., 2009; Zhou et al., 2013). These inconsistencies across studies may have been influenced by the variability of sample characteristics and differences in the methodology. However, we think that our study is one of the very few longitudinal studies on this topic, which provides solid evidence in the assessment of asymmetry changes. Thus, based on our results, we conclude that the anterior and the posterior language-related regions present opposite patterns of cortical thickness asymmetry, that is the frontal and temporal regions are left- and right-lateralized, respectively.

To further investigate whether cortical thickness asymmetry changes with age, we examined the changes between the ages of 5 and 6 years. Although a similar asymmetry pattern for both ages was observed, we found longitudinal asymmetry changes in the HG (generally showing a trend towards rightward asymmetry at both time points), which became less right-lateralized within the one-year-interval (i.e., between 5 and 6 years). However, we did not find longitudinal asymmetry changes in frontal regions. That cortical thickness asymmetry evolves dynamically across development, is consistent with most of the previous studies (Li et al., 2015; Plessen et al., 2014; Shaw et al., 2009; Zhou et al., 2013), but not all studies (Koelkebeck et al., 2014). However, in contrast to the prominent changes observed in previous research, we only found significant changes of cortical thickness asymmetry in HG. Given that longitudinal findings are bound to the start- and end-points of analyses, the selection of temporal intervals, and to the number of time points in the analyses (Brandmaier et al., 2015; King et al., 2018; Kraemer et al., 2000; Rast and Hofer, 2014), we suppose that the age range and the narrow scanning intervals between time points in the present study might have contributed to these discrepancies with other studies. Specifically, all above-mentioned studies were conducted in samples with large age ranges (Kong et al., 2018; Plessen et al., 2014; Shaw et al., 2009; Zhou et al., 2013) and approximately 2–5 years between each testing time point (Lu et al., 2007; Shaw et al., 2009; Sowell, 2004). Thus, the minimal changes in structural asymmetry found in our sample of children might be attributed to the lack of measurable change at group-level across a short time period (i.e., 1-year-interval). However, and more importantly, the observed large individual differences in structural asymmetry (also in language performance) enabled us to further examine individual differences of brain asymmetry changes and the association to language performance changes (Lindenberger, 2014).

### 4.3 Cortical thickness asymmetry changes and language ability changes across development

Our findings on the relation between cortical thickness asymmetry changes (between ages 5 and 6) and language ability changes (between ages 5 and 6) demonstrated that a stronger increase in sentence comprehension abilities was associated with larger cortical thinning in the left triangular part of the IFG compared to the right IFG. In line, the acquisition of cognitive abilities, such as language, was shown to be related to rapid cortical thinning (Shaw 2009). Extending previous literature showing a thinner cortex in left inferior frontal regions to be associated with enhanced performance in phonological and vocabulary tasks in 5- to 11-year-olds (Lu et al., 2007; Sowell, 2004), we showed that greater cortical thinning in the left compared to the right triangular part of the IFG is related to an increase in sentence comprehension abilities between the ages of 5 and 6 years. Thus, a more mature pattern of the left triangular part of the IFG (i.e., greater cortical thinning the left triangular part of the IFG) in comparison to the right seems to be related to higher language abilities (here: higher sentence comprehension abilities). This is in line with asymmetry reported in a previous diffusion study (Lebel and Beaulieu, 2009) and a functional study (Groen et al., 2012) and forms the basis for the assumption of the IFG to be one of the core language regions (see also Friederici, 2011; Knoll et al., 2012). The assumption of the importance of the IFG for language functions is further supported by our finding that a relatively thinner cortical thickness in the left compared to the right IFG in 5- and 6-year-old children is predictive for higher sentence comprehension abilities at the age of 7 years. Thus, we suggest that changes in sentence comprehension abilities are associated with the cortical thickness asymmetry changes in the inferior frontal regions, highlighting the IFG’s crucial role in language acquisition and sentence comprehension.

In addition, asymmetry changes (between ages 5 and 6) showed a trend to correlate (correlations did not survive correction for multiple comparison) with language ability changes (between ages 6 and 7) in temporal regions, i.e., the STS and the TP, suggesting that greater rightward asymmetry in the STS and the TP in children from 5 to 6 years tend to show a greater improvement in sentence comprehension from 6 to 7 years. Despite the weak effect, this finding is in line with previous studies identifying the left IFG, together with the left superior temporal cortex, as the language comprehension network (den Ouden et al., 2012; Friederici, 2012). Together, our findings might indicate that the asymmetry of the left fronto-temporal language comprehension network, particularly the IFG, is associated with sentence comprehension and its development.

### 4.4 Limitations and future directions

Several limitations of the present study should be considered. First, although longitudinal models can be easily fitted to samples with relatively small sample size (e.g., 22 subjects for structural equation modeling) (Curran et al., 2010; Kievit et al., 2018), we need to point out that our findings are only based on a modest sample size of 50 children and should therefore be treated with caution. Results should be verified in future studies using larger samples to obtain more solid inferences. Furthermore, as discussed before, our findings only present subtle asymmetry changes across time, suggesting a lack of changes at the average group-level. We suggest to optimize the design interval and collect more time point measurements in future longitudinal studies to obtain measurable brain changes and reliable developmental trajectories across time. Moreover, other language capacities, such as phonological skills and vocabulary scores, should be tested to provide a more comprehensive understanding of the relationship between structural asymmetry and language abilities. Finally, to exclude the impact of possible atypical asymmetry (hemispheric dominance) in left-handers (i.e., 30% have the atypical asymmetry), we only included right-handers (i.e., over 90% of them have the typical asymmetry) (Guadalupe et al., 2014; Knecht, 2000). However, the mere inclusion of right-handers might result in the found left-hemispheric bias for language abilities (Shaw et al., 2009), which should be considered by including a balanced number of left- and right-handers in future studies to cancel out this sample selection bias.

## 5 Conclusion

Taken together, we could demonstrate that children’s language abilities as well as language-related cortical thickness asymmetry change between the ages of 5 and 6 years. Specifically, we showed that the asymmetry of the language core region, namely IFG, is associated with the children’s language performance. Five- and six-year-old children with greater cortical thinning in the left triangular part of the IFG showed better sentence comprehension abilities when they were 7 years old. More intriguingly, longitudinal cortical thickness asymmetry changes of the triangular part of the IFG were also related to sentence comprehension improvement in children across development. To our knowledge, our longitudinal study is the first to show the association between structural asymmetry and language abilities across development. Further, it uncovers the coupling changes between brain and language abilities, and further highlights the crucial role of the IFG in language development.
