# Supplementary material for: Cortical thickness lateralization and its relation to language abilities in children
Source: Dev Cogn Neurosci. 2019 Aug 22;39:100704. doi: 10.1016/j.dcn.2019.100704 (PMC6892251; doi:10.1016/j.dcn.2019.100704)
Supplement: Supplementary file 1 [file mmc1.docx]

**Supplementary materials of the manuscript “Cortical thickness lateralization and its relation to language abilities in children”**

1. **Validation of regions of interest (ROIs) selection**

Using NeuroSynth (Yarkoni et al., 2011), a term-based meta-analysis approach, (<http://neurosynth.org/>), we identified brain mappings with the term “sentence comprehension, language comprehension, and language network to verify our selection of regions of interest (ROIs). As shown in Figure S2, the resulting term-based language networks mainly included the inferior frontal, superior, and middle temporal regions. Of note, precentral gyrus, known as the primary motor cortex, probably subserving as the sensory language region was also found in the meta-analysis. However, we decided not to include it given that we were aiming to concentrate on the core language comprehension regions in the present study. We additionally included temporo-parietal areas (i.e., inferior parietal and supramarginal gyrus), because they were reported to be involved in sentence comprehension in a previous meta-analysis (Vigneau et al., 2006).

1. **Regional-wise and vertex-wise cortical thickness asymmetry for the whole brain**

Aside from the selected 11 ROIs (see main text), we also examined the regional-wise cortical thickness asymmetry of the entire brain (see Figure S3 A and B, and Table S2) with a significance level of *p* < 0.002 (i.e., 0.05/34 regions, Bonferroni corrected) to gain a complete overview of the cortical thickness asymmetry for the entire brain. At age 5, anterior regions including orbital, postcentral, middle and superior frontal gyrus, and cingulate gyrus were significantly left-lateralized; while posterior regions including cuneus, lateral occipital, lingual and temporal pole were significantly right-lateralized (*p* < 0.002, Bonferroni corrected). Similar lateralization was also observed in children at age 6. Importantly, these regional-wise asymmetry patterns were replicated when performing the vertex-wise analysis as described below (see Figure S3 C and D, and Table S2).

1. **Validation of vertex-based analysis**

A vertex-wise analysis, which improves the spatial resolution and reduces the potential influences from the specific parcellation, as used for the results in the main manuscript, was conducted for validation purposes. We examined the changes of cortical thickness asymmetry and the association with language development equivalently to the regional-wise analysis presented in the main manuscript.

**3.1 Data preprocessing**

After the preprocessing procedures mentioned in the main text, we further ran the *xhemi* procedure as the default procedures for symmetric analysis in Freesurfer (Greve et al., 2013). The vertex-wise cortical thickness maps of individuals were aligned to the common surface-based symmetric and unbiased template, namely *fsaverage_sym*, implemented in Freesurfer to avoid the hemispheric bias of the default common template *fsaverage*. Finally, both left and right hemispheric maps were projected to the left hemisphere of the *fsaverage_sym* for each individual, resulting in one-to-one mapped vertices across hemispheres. Finally, thickness maps were smoothed using a 20-mm full-width-at-half-maximum (FWHM) Gaussian Kernel.

**3.2 Statistical analysis**

The Surfstat toolbox (http://www.math.mcgill.ca/keith/surfstat/) was used for the vertex-wise analyses. For all models, a fixed effect of time and a random effect of subject were examined, and baseline age (i.e., age at first testing time point), and gender were controlled for by including them as covariates of no interest. Meanwhile, non-verbal IQ, together with the covariates mentioned above, was included as a covariate of no interest when performing analyses concerning language abilities. To assess the relationship between brain asymmetry and language performance, we correlated brain lateralization with language performance. Specifically, to examine how changes in brain asymmetry co-vary with changes in sentence comprehension abilities, the difference scores (i.e., brain asymmetry and sentence comprehension abilities changes) were calculated by subtracting the measures at the first time point from the measures at the second time point. Significance values of all surface-based results were corrected by false discovery ratio (FDR) for multiple comparisons with a significance level of *p* < 0.05.

**3.3 Results and discussion**

We did not find significant cortical thickness asymmetry changes at the whole brain level in children between 5 and 6 years of age, suggesting that there were no measurable asymmetry changes at group-level over a short time. This finding was consistent with our regional findings, where asymmetries of most of the language-related ROIs did not change significantly across development.

In a next step, we examined whether cortical thickness asymmetry changes were associated with language changes in children between 5 and 6 years of age and found a negative correlation in the inferior frontal gyrus (IFG, *p* < 0.05, FDR-corrected), including the triangular and opercular part of the IFG (see Figure S4). This finding is in line with our regional findings and survived after correcting for multiple comparisons even at the whole brain level, suggesting that children with larger language improvements show greater cortical thinning in the left IFG compared to the right.

In addition, we also found a negative correlation (*p* < 0.05, FDR-corrected) between the early cortical thickness asymmetry (at age 5 years) and later language abilities (at age 7 years) in the middle frontal gyrus (MFG), and supramarginal gyrus (see Figure S5). These findings indicate that children with greater cortical thinning in the left frontal regions and the left supramarginal gyrus (in comparison to the right counterparts) at age 5 showed higher sentence comprehension abilities when they were 7 years. In addition, the negative correlation was not revealed in the brain of children at age 6 years similar to the regional findings. The slight inconsistencies were likely caused by the different methodological approaches applied.

**Tables**

Table S1. *Comparison between linear mixed models with and without random slope.*

|  | Model | df | AIC | BIC | logLik | L.Ratio | *p-values* |
| --- | --- | --- | --- | --- | --- | --- | --- |
| M1 | 1 | 9 | 754.04 | 781.60 | -368.02 |  |  |
| M2 | 2 | 11 | 757.14 | 790.82 | -367.57 | 0.91 | 0.636 |

*^Note.^* ^M1 is the reference model, including time as a fixed effect, subject as a random effect, autoregressive covariance structure (AR1) and covariates of no interest (i.e., baseline age, gender, non-verbal IQ, handedness). Random slope was further entered in the model as M2, which did not improve the model significantly.^

Table S2. *Cortical thickness asymmetry of language-related ROIs and the remaining brain regions from the Desikan-Killiany atlas at first and second testing time points.*

|  | T1 | |  | T2 |  | |
| --- | --- | --- | --- | --- | --- | --- |
| Regions | *t values* | *uncorrected p* | *effect size* | *t values* | *uncorrected p* | *effect size* |
| *Language-related ROIs* | | | | | | |
| Bankssts | -2.01 | 0.049 | 0.27 | -1.58 | 0.119 | 0.21 |
| Caudalmiddlefrontal | 2.89 | 0.007 | 0.38 | ***3.87*** | ***< 0.001*** | ***0.52*** |
| Inferiorparietal | -1.60 | 0.115 | 0.21 | -1.84 | 0.071 | 0.25 |
| Middletemporal | -2.55 | 0.014 | 0.34 | **-2.98** | **0.004** | **0.40** |
| Parsopercularis | 2.40 | 0.020 | 0.32 | 2.58 | 0.013 | 0.35 |
| Parsorbitalis | **3.21** | **0.002** | **0.43** | 2.70 | 0.009 | 0.36 |
| Parstriangularis | 1.19 | 0.239 | 0.16 | 1.10 | 0.278 | 0.15 |
| Superiortemporal | -2.65 | 0.010 | 0.35 | -2.68 | 0.010 | 0.36 |
| Supramarginal | 2.17 | 0.034 | 0.29 | 2.65 | 0.010 | 0.36 |
| Temporalpole | ***-4.89*** | ***< 0.001*** | ***0.65*** | ***-5.25*** | ***<0.001*** | ***0.70*** |
| Transversetemporal | -1.47 | 0.147 | 0.20 | -0.15 | 0.882 | 0.02 |
|  |  |  |  |  |  |  |
| *Non-language-related brain regions* | | | | | | |
| Caudalanteriorcingulate | *7.26* | *< 0.001* | *0.970* | *7.14* | *< 0.001* | *0.954* |
| Cuneus | *-5.22* | *< 0.001* | *0.698* | *-4.79* | *< 0.001* | *0.640* |
| Entorhinal | -3.28 | 0.002 | 0.438 | *-3.51* | *< 0.001* | *0.469* |
| Fusiform | -1.72 | 0.091 | 0.230 | -1.11 | 0.271 | 0.149 |
| Inferiortemporal | 1.95 | 0.057 | 0.260 | 1.74 | 0.087 | 0.233 |
| Isthmuscingulate | -1.73 | 0.090 | 0.231 | -1.87 | 0.066 | 0.250 |
| Lateraloccipital | *-6.72* | *< 0.001* | *0.898* | *-6.28* | *< 0.001* | *0.839* |
| Lateralorbitofrontal | *3.73* | *< 0.001* | *0.499* | 2.55 | 0.014 | 0.341 |
| Lingual | *-3.51* | *0.001* | *0.469* | -3.02 | 0.004 | 0.404 |
| Medialorbitofrontal | -1.76 | 0.084 | 0.235 | -1.03 | 0.309 | 0.137 |
| Parahippocampal | 2.50 | 0.015 | 0.334 | 2.57 | 0.013 | 0.343 |
| Paracentral | 2.73 | 0.008 | 0.365 | 1.81 | 0.076 | 0.242 |
| Pericalcarine | -0.63 | 0.530 | 0.084 | -0.44 | 0.661 | 0.059 |
| Postcentral | *3.51* | *0.001* | *0.469* | 3.32 | 0.002 | 0.443 |
| Posteriorcingulate | *4.26* | *< 0.001* | *0.569* | *3.81* | *< 0.001* | *0.509* |
| Precentral | 3.13 | 0.003 | 0.418 | *3.61* | *< 0.001* | *0.482* |
| Precuneus | -0.33 | 0.743 | 0.044 | -0.37 | 0.715 | 0.049 |
| Rostralanteriorcingulate | 1.72 | 0.092 | 0.229 | 1.45 | 0.152 | 0.194 |
| Rostralmiddlefrontal | *6.47* | *< 0.001* | *0.864* | *5.83* | *< 0.001* | *0.780* |
| Superiorfrontal | *5.93* | *< 0.001* | *0.792* | *6.83* | *< 0.001* | *0.913* |
| Superiorparietal | 2.63 | 0.011 | 0.351 | 3.04 | 0.004 | 0.406 |
| Frontalpole | 1.13 | 0.261 | 0.152 | 0.91 | 0.368 | 0.121 |
| Insula | -0.80 | 0.426 | 0.107 | -1.56 | 0.125 | 0.208 |

*^Note.^* ^Bold indicates statistical significance with a^ *^p-value^* ^below 0.05 after Bonferroni correction (i.e., uncorrected^ *^p-values^* ^< 0.005) for language-related ROIs. In addition, italic indicates statistical significance with a^ *^p-value^* ^below 0.05 after Bonferroni correction (i.e., uncorrected^ *^p-values^* ^< 0.05/34 = 0.002) for the entire brain regions (also see Figure S3). T1 indicates the first testing point and T2 indicates the second testing point.^

Table S3. *Longitudinal cortical thickness asymmetry changes across development.*

|  | Fixed effect | | | | | Random effect | | |
| --- | --- | --- | --- | --- | --- | --- | --- | --- |
| Regions | *estimate* | *t* | *p* | *CI*  *(lower)* | *CI*  *(upper)* | *SD* | *CI*  *(lower)* | *CI*  *(upper)* |
| Bankssts | 0.002 | 1.41 | 0.165 | -0.001 | 0.005 | 0.03 | **0.029** | **0.041** |
| Caudalmiddlefrontal | 0.003 | 1.68 | 0.109 | -0.001 | 0.006 | 0.02 | **0.013** | **0.020** |
| Inferiorparietal | -0.000 | -0.37 | 0.712 | -0.002 | 0.002 | 0.02 | **0.012** | **0.018** |
| Middletemporal | -0.001 | -0.44 | 0.662 | -0.003 | 0.002 | 0.02 | **0.018** | **0.022** |
| Parsopercularis | 0.001 | 0.37 | 0.712 | 0.000 | 0.003 | 0.02 | **0.016** | **0.023** |
| Parsorbitalis | -0.003 | -1.40 | 0.166 | -0.008 | 0.001 | 0.04 | **0.033** | **0.049** |
| Parstriangularis | -0.000 | -0.09 | 0.926 | -0.004 | 0.004 | 0.02 | **0.020** | **0.030** |
| Superiortemporal | 0.000 | 0.30 | 0.767 | -0.002 | 0.003 | 0.02 | **0.016** | **0.024** |
| Supramarginal | 0.002 | 0.98 | 0.331 | -0.002 | 0.005 | 0.02 | **0.019** | **0.027** |
| Temporalpole | -0.004 | -1.58 | 0.121 | -0.000 | 0.001 | 0.03 | **0.023** | **0.035** |
| **Transversetemporal** | **0.008** | **3.15** | **0.003** | **0.003** | **0.013** | **0.041** | **0.034** | **0.050** |

*^Notes.^* ^Bold indicates statistical significance. The asymmetry of the transverse temporal gyrus [known as heschl's gyrus (HG)] showed significant changes across the different time points. The intercept of the random effect was significant for all ROIs.^

Table S4. *Correlations between asymmetry changes and language abilities changes across development.*

| Regions | *r* | *uncorrected p-values* |
| --- | --- | --- |
| *Language-related ROIs* | | |
| Bankssts | -0.18 | 0.222 |
| Caudalmiddlefrontal | -0.21 | 0.157 |
| Inferiorparietal | -0.26 | 0.082 |
| Middletemporal | 0.11 | 0.469 |
| Parsopercularis | -0.17 | 0.244 |
| Parsorbitalis | -0.37 | 0.012 |
| ***Parstriangularis*** | ***-0.45*** | ***0.002*** |
| Superiortemporal | -0.05 | 0.766 |
| Supramarginal | -0.11 | 0.480 |
| Temporalpole | 0.13 | 0.374 |
| Transversetemporal | 0.18 | 0.229 |
|  |  |  |
| *Non-language-related brain regions* | | |
| Caudalanteriorcingulate | -0.09 | 0.542 |
| Cuneus | -0.24 | 0.112 |
| Entorhinal | 0.14 | 0.351 |
| Fusiform | 0.02 | 0.892 |
| Inferiortemporal | 0.08 | 0.595 |
| Isthmuscingulate | 0.08 | 0.596 |
| Lateraloccipital | -0.02 | 0.900 |
| Lateralorbitofrontal | -0.31 | 0.037 |
| Lingual | 0.17 | 0.264 |
| Medialorbitofrontal | -0.31 | 0.034 |
| Parahippocampal | 0.17 | 0.245 |
| Paracentral | -0.14 | 0.357 |
| Pericalcarine | -0.12 | 0.438 |
| Postcentral | -0.13 | 0.374 |
| Posteriorcingulate | 0.13 | 0.391 |
| Precentral | 0.11 | 0.466 |
| Precuneus | -0.16 | 0.290 |
| Rostralanteriorcingulate | -0.06 | 0.690 |
| Rostralmiddlefrontal | -0.25 | 0.091 |
| Superiorfrontal | -0.34 | 0.018 |
| Superiorparietal | -0.17 | 0.251 |
| Frontalpole | 0.21 | 0.157 |
| Insula | 0.05 | 0.724 |

*^Notes.^* ^Bold indicates statistical significance with a^ *^p-value^* ^below 0.005 (i.e., 0.05/11 ROIs) after multiple comparison correction for language-related ROIs. The asymmetry changes in the triangular part of the IFG were significantly correlated with the language abilities changes across development. In addition, the orbital part of the IFG showed a trend level of correlation with language abilities changes with an uncorrected^ *^p-value^* ^of 0.012 < 0.05. Moreover, italic indicates statistical significance with a^ *^p-value^* ^below 0.002 (i.e., 0.05/34 ROIs) after multiple comparison correction for all brain regions. The asymmetry changes in the triangular part of the IFG were significantly correlated with the language abilities changes across development. Of note, covariates of no interest (i.e., baseline age, gender, handedness, non-verbal IQ) were controlled for when performing the correlation analyses.^

Table S5. *Correlations between the brain’s asymmetry in children at age 5 and at age 6 and later language abilities at age 7.*

| Region | Asymmetry at age 5 | | Asymmetry at age 6 | |
| --- | --- | --- | --- | --- |
|  | *r* | *uncorrected* *p-values* | *r* | *uncorrected* *p-values* |
| *Language-related ROIs* | | |  |  |
| Bankssts | 0.07 | 0.637 | 0.00 | 0.975 |
| Caudalmiddlefrontal | -0.00 | 0.976 | -0.06 | 0.677 |
| Inferiorparietal | 0.10 | 0.513 | 0.04 | 0.788 |
| Middletemporal | 0.05 | 0.732 | -0.01 | 0.925 |
| Parsopercularis | 0.02 | 0.899 | 0.05 | 0.764 |
| Parsorbitalis | -0.26 | 0.081 | -0.23 | 0.113 |
| **Parstriangularis** | **-0.40** | **<0.005** | **-0.42** | **0.003** |
| Superiortemporal | -0.04 | 0.769 | -0.12 | 0.426 |
| Supramarginal | 0.03 | 0.841 | 0.00 | 0.982 |
| Temporalpole | -0.07 | 0.642 | -0.22 | 0.141 |
| Transversetemporal | 0.18 | 0.225 | 0.13 | 0.378 |
|  |  |  |  |  |
| *Non-language-related brain regions* | | |  |  |
| Caudalanteriorcingulate | 0.16 | 0.283 | 0.06 | 0.674 |
| Cuneus | 0.15 | 0.313 | 0.11 | 0.476 |
| Entorhinal | -0.29 | 0.045 | -0.28 | 0.056 |
| Fusiform | 0.12 | 0.439 | 0.05 | 0.738 |
| Inferiortemporal | -0.04 | 0.806 | -0.07 | 0.624 |
| Isthmuscingulate | 0.04 | 0.799 | 0.08 | 0.597 |
| Lateraloccipital | 0.15 | 0.300 | 0.14 | 0.349 |
| Lateralorbitofrontal | -0.13 | 0.383 | -0.08 | 0.607 |
| Lingual | -0.08 | 0.609 | -0.03 | 0.834 |
| Medialorbitofrontal | 0.06 | 0.702 | 0.25 | 0.088 |
| Middletemporal | 0.05 | 0.732 | -0.01 | 0.925 |
| Parahippocampal | -0.07 | 0.659 | -0.10 | 0.486 |
| Paracentral | 0.24 | 0.100 | 0.23 | 0.124 |
| Pericalcarine | 0.24 | 0.098 | 0.23 | 0.114 |
| Postcentral | 0.19 | 0.205 | 0.10 | 0.520 |
| Posteriorcingulate | 0.17 | 0.258 | 0.26 | 0.083 |
| Precentral | -0.00 | 0.976 | -0.02 | 0.872 |
| Precuneus | -0.01 | 0.942 | 0.07 | 0.651 |
| Rostralanteriorcingulate | -0.21 | 0.149 | -0.14 | 0.343 |
| Rostralmiddlefrontal | -0.07 | 0.620 | -0.11 | 0.447 |
| Superiorfrontal | -0.16 | 0.271 | -0.09 | 0.562 |
| Superiorparietal | 0.04 | 0.778 | -0.02 | 0.906 |
| Frontalpole | 0.07 | 0.621 | 0.03 | 0.863 |
| Insula | -0.14 | 0.360 | -0.08 | 0.581 |

*^Notes.^* ^Bold indicates statistical significance with a^ *^p-value^* ^below 0.005 (i.e., 0.05/11 ROIs) after multiple comparison correction for language-related ROIs. Of note, covariates of no interest (i.e., baseline age, gender, handedness, non-verbal IQ) were controlled for when performing the correlation analyses.^

Table S6. *Cortical thickness and its changes across time for brain regions for which significant effects can be reported*

| Region | T1 | | T2 | | ΔT | *p* | *t* |
| --- | --- | --- | --- | --- | --- | --- | --- |
|  | *mean* | *SD* | *mean* | *SD* |  |  |  |
| L_IFGtri | 2.967 | 0.143 | 2.946 | 0.158 | -0.021 | 0.058 | 1.938 |
| R_IFGtri | 2.941 | 0.120 | 2.922 | 0.135 | -0.020 | 0.149 | 1.464 |
| L_HG | 2.873 | 0.238 | 2.871 | 0.240 | -0.001 | 0.920 | 0.101 |
| R_HG | 2.928 | 0.269 | 2.878 | 0.258 | -0.049 | <0.001 | 4.294 |

*^Note.^* ^T1 indicates cortical thickness at the first testing time point, T2 indicates cortical thickness at the second testing time point, and ΔT indicates changes of cortical thickness across time (i.e., T2-T1). IFGtri = triangular part of the IFG. Cortical thinning was observed in both the triangular IFG and the HG, but a greater thinning in the left IFG and the right HG compared to their counterparts. It further confirmed that the significant correlation between asymmetry changes and language ability changes in the triangular part of the IFG (see result 3.3) was driven by greater thinning in the left IFG in comparison to the right IFG. Likewise, the greater leftward asymmetry in the HG across development (see result 3.2) can be attributed to the greater thinning in the right HG compared to the left.^

**Figures**

**
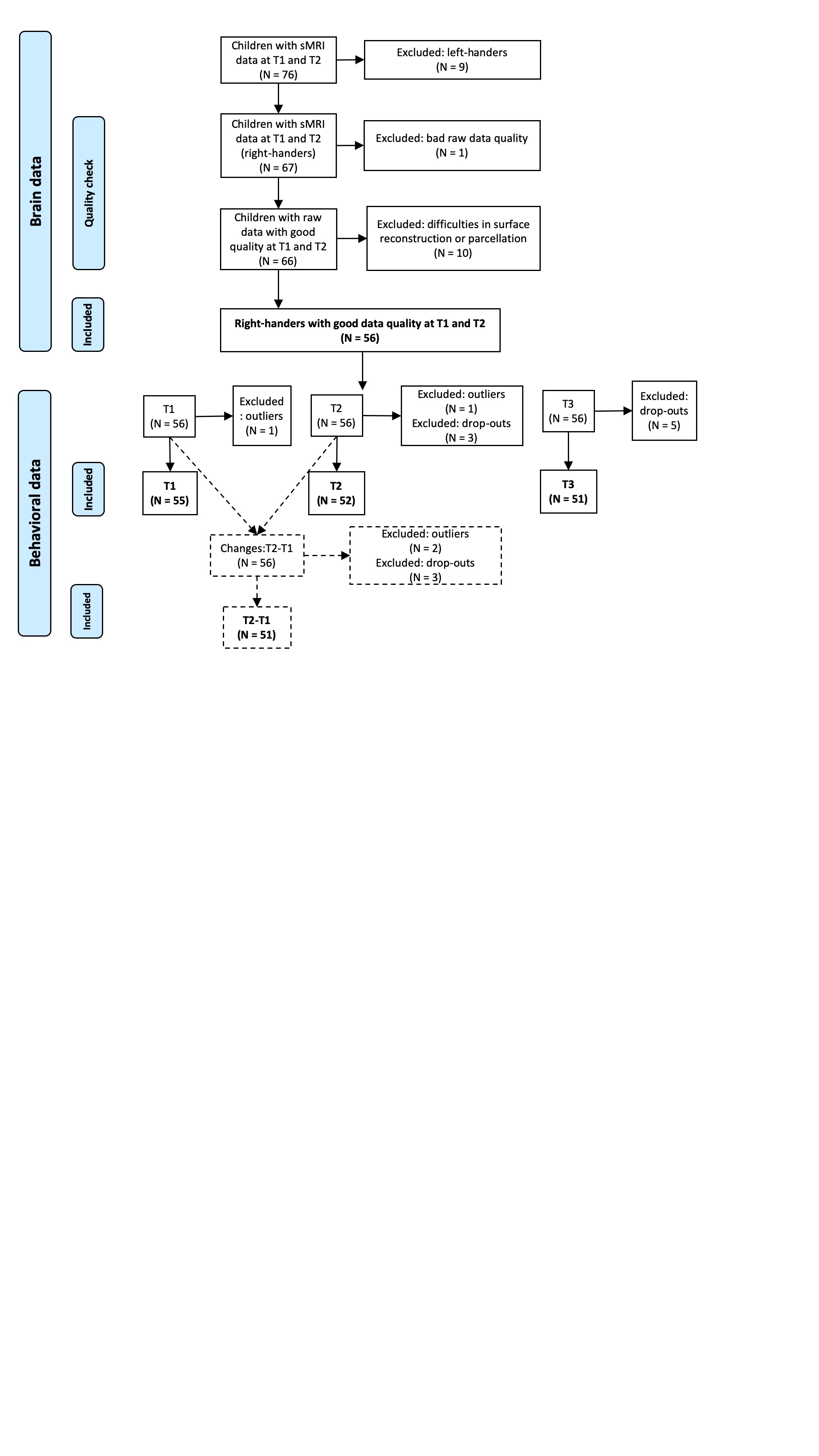
**

*Figure S1.* Flow chart for the sample inclusion concerning the brain and behavioral data across the three testing time points. Bold indicates the sample included in the final analyses. Dashed frames and arrows indicate changes in language performance from ages 5 to 6 years. T1, T2, and T3 denote the first, second, and third testing time point.

**
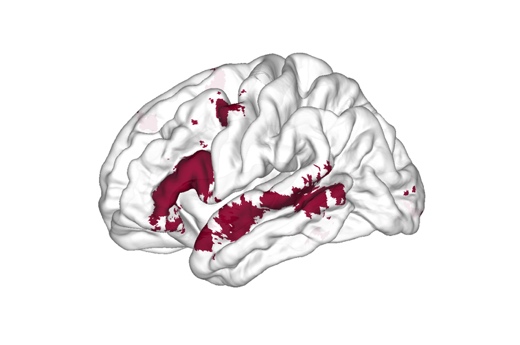
**

*Figure S2.* Region of interest (ROI) selection verified from term-based meta-analysis. ROIs include inferior and middle frontal, superior temporal, middle temporal regions, as well as the precentral gyrus.


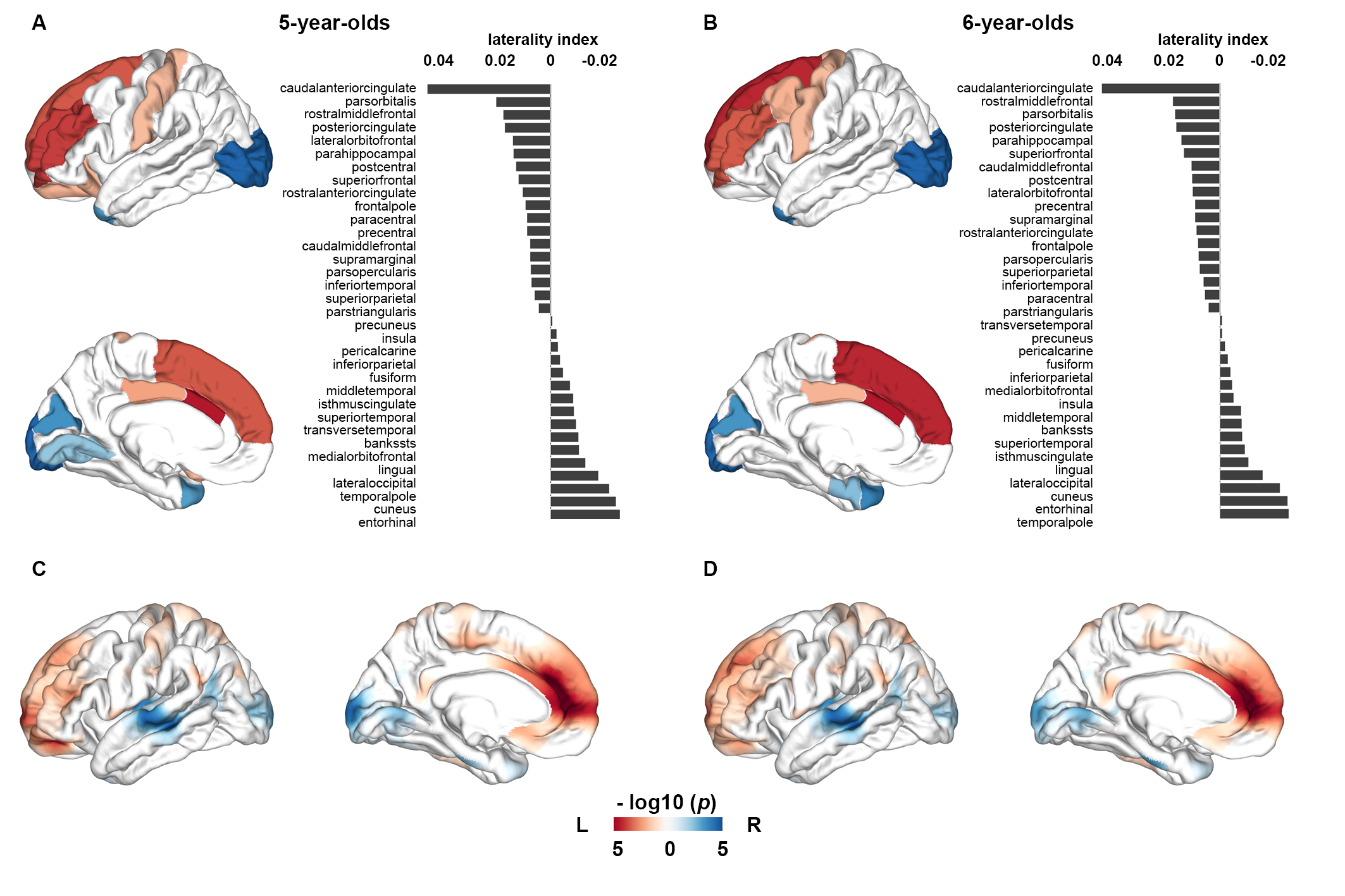


*Figure S3.* Cortical thickness asymmetry in the entire brain in children aged 5 years (A, C) and 6 years (B, D). A and B indicate regional-wise cortical thickness asymmetry patterns; C and D indicate the vertex-wise cortical thickness asymmetry patterns. A positive laterality index indicates leftward asymmetry, while a negative laterality index indicates rightward asymmetry. Regions colored in red denote significant left-lateralized regions, and regions in blue denote significant right-lateralized regions. For visualization purposes, the corrected *p*-value was log-transformed, and the darker color indicates more significant *p-*value. L, left; R, right.

**
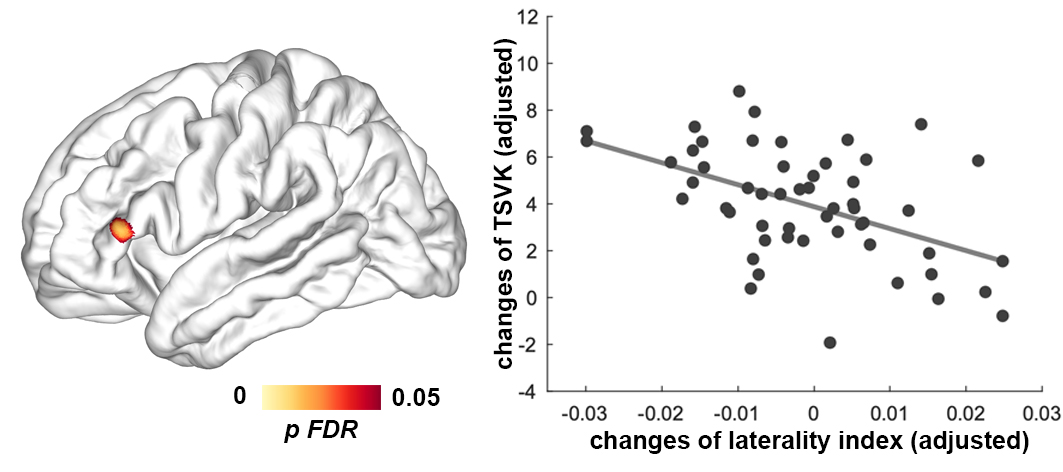
**

*Figure S4*. Illustrated is the negative correlation between cortical thickness asymmetry changes in the IFG and changes of language performance in children between the ages of 5 and 6 years, after the adjustment for covariates of no interest (i.e., baseline age, gender, handedness, non-verbal IQ).


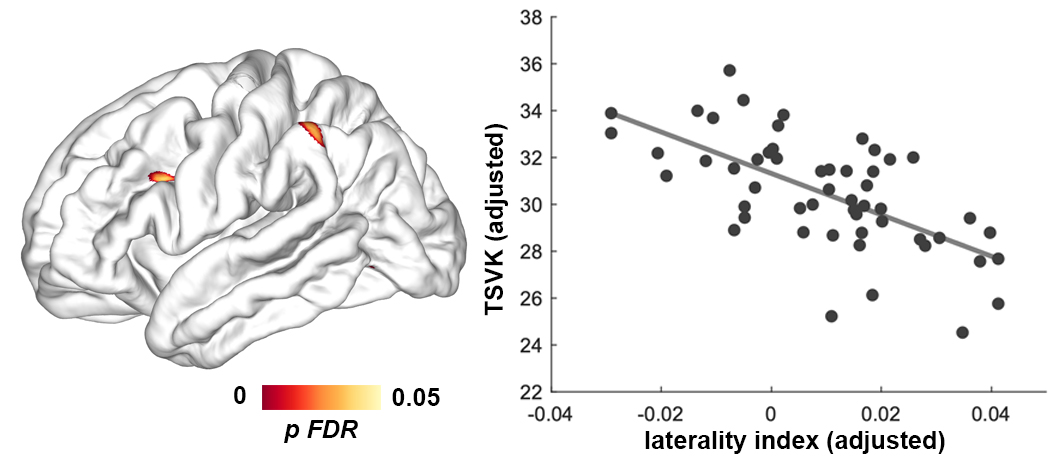


*Figure S5.* Illustrated is the negative correlation between early cortical asymmetry (at age 5 years) and later language performance in children at age 7 years in the middle frontal and supramarginal gyrus, after the adjustment for covariates of no interest (i.e., baseline age, gender, handedness, non-verbal IQ).

**References**

Greve, D.N., Van der Haegen, L., Cai, Q., Stufflebeam, S., Sabuncu, M.R., Fischl, B., Brysbaert, M., 2013. A Surface-based Analysis of Language Lateralization and Cortical Asymmetry. J. Cogn. Neurosci. doi:10.1162/jocn_a_00405

Kong, X.-Z., Mathias, S.R., Guadalupe, T., Glahn, D.C., Franke, B., Crivello, F., Tzourio-Mazoyer, N., Fisher, S.E., Thompson, P.M., Francks, C., 2018. Mapping cortical brain asymmetry in 17,141 healthy individuals worldwide via the ENIGMA Consortium. Proc. Natl. Acad. Sci. doi:10.1073/pnas.1718418115

Vigneau, M., Beaucousin, V., Hervé, P.Y., Duffau, H., Crivello, F., Houdé, O., Mazoyer, B., Tzourio-Mazoyer, N., 2006. Meta-analyzing left hemisphere language areas: Phonology, semantics, and sentence processing. Neuroimage. doi:10.1016/j.neuroimage.2005.11.002

Yarkoni, T., Poldrack, R.A., Nichols, T.E., Essen, D.C. Van, Wager, T.D., 2011. NeuroSynth: a new platform for large-scale automated synthesis of human functional neuroimaging data. Front. Neuroinform. 5. doi:10.3389/conf.fninf.2011.08.00058
